# Monocyte chemotaxis in bronchial carcinoma and cigarette smokers.

**DOI:** 10.1038/bjc.1977.215

**Published:** 1977-10

**Authors:** A. B. Kay, J. G. McVie

## Abstract

Chemotaxis of blood monocytes was measured in 31 patients with bronchial carcinoma and 19 cigarette smokers. Thirteen patients with metastatic bronchial carcinoma had significantly less (P less than 0.005) chemotactic response than matched controls. Those with disease confined to the chest, or with recurrent or operable bronchial carcinoma, had no significant depression of monocyte chemotaxis. There was also no significant difference in monocyte chemotaxis between cigarette smokers and matched controls. These results support the concept that in human cancer there is a defect in monocyte chemotaxis, but in bronchial carcinoma significant depression was only apparent in those with advanced disease.


*Br. J. Cancer* (1977) **36**, 461.

### MONOCYTE CHEMOTAXIS IN BRONCHIAL CARCINOMA AND CIGARETTE SMOKERS

A. B. KAY* AND J. G. McVIE†

*From the *Departments of Respiratory Diseases and Pathology, University of Edinburgh, and † Department of Clinical Oncology, University of Glasgow, Gartnavel General Hospital, Glasgow*

Received 9 May 1977 Accepted 13 June 1977

**Summary.—Chemotaxis of blood monocytes was measured in 31 patients with bronchial carcinoma and 19 cigarette smokers. Thirteen patients with metastatic bronchial carcinoma had significantly less ($P < 0{\cdot}005$) chemotactic response than matched controls. Those with disease confined to the chest, or with recurrent or operable bronchial carcinoma, had no significant depression of monocyte chemotaxis. There was also no significant difference in monocyte chemotaxis between cigarette smokers and matched controls. These results support the concept that in human cancer there is a defect in monocyte chemotaxis, but in bronchial carcinoma significant depression was only apparent in those with advanced disease.**

THE role of cells of the mononuclear phagocytic series in immune surveillance has been suggested by a number of workers (Hibbs, Lambert and Remington, 1972; Alexander, 1976). For example, an inhibitor of macrophage chemotaxis produced by various transplanted tumours in mice has been described (Snyderman and Pike, 1976) and in man the capacity of peripheral blood monocytes to respond by chemotaxis *in vitro* was depressed in patients with genito-urinary neoplasms (Hausman *et al.*, 1975), malignant melanoma (Rubin, Cosimi and Goetzl, 1976) and other human cancers (Boetcher and Leonard, 1974). We have studied monocyte chemotaxis from 31 patients with bronchial carcinoma at various clinical stages, and also the monocyte chemotactic response of cigarette smokers who are known to be at risk for developing bronchial neoplasms.

### PATIENTS AND CONTROLS

Patients with bronchial carcinoma were classified according to the stage of their disease. Those with small tumours deemed suitable for surgical resection were termed *operable*. Disease which reappeared locally at the site of a surgical resection was termed *recurrent*. Disease *confined to the chest* had spread locally from the primary site in the bronchus to involve surrounding lung, local lymph nodes and chest wall. The *metastatic* group had deposits of tumour outside the chest, commonly in liver or bone, as demonstrated clinically, or by radionuclide scanning.

Controls for the cancer groups were all convalescent, hospitalized patients who had sustained either myocardial infarctions or respiratory infections and in whom there was no evidence of malignant disease. Controls for cigarette smokers were all healthy, non-smoking volunteers.

### MATERIALS AND METHODS

Human peripheral-blood monocytes were separated on a Ficoll–Triosil gradient as previously described (Böyum, 1968). Chemotaxis was quantified either by the "leading front" method using Millipore filters (Millipore Co., Wembley) of 8 μm pore size (Zigmond and Hirsch, 1973) or by the method of Snyderman *et al.*, (1972) employing

Correspondence: Dr A. B. Kay, Department of Pathology, University of Edinburgh, Medical School, Teviot Place, Edinburgh, EH8 9AG.

Nucleopore filters and polycarbonate "Boyden chambers" Neuroprobe, Bethesda, Maryland, U.S.A.). The only modification was that the suspending medium for monocytes and for dilutions of chemoattractant was Medium 199 containing 30 mM Hepes buffer. The chemoattractant was either human serum in which the complement system had been activated with purified cobra venom factor (CVF) (Ballow and Cochrane, 1969) or solutions of casein (British Drug Houses). Casein was used in the early part of the study, but its chemoattracting properties often deteriorated after a few days, even under a variety of storage conditions. The experiments with casein reported here are with freshly prepared material. In contrast, serum activated with CVF was divided into portions after preparation and stored at −80°C until use.

The chemotactic responses from patients with bronchial carcinoma or cigarette smokers were compared with age- and sex-matched controls and each pair was performed on the same day under the same experimental conditions. A three-point dose-response of chemoattractant was performed for each experiment. Optimal monocyte migration of cells, either from patients or smokers, was achieved with 0·5 mg/ml of fresh casein or 2·5% CVF-activated serum. Each assay was performed in duplicate, and measurements from each filter were the pooled results from 10 random high-power fields. The test and control samples were analysed by the Wilcoxon test of paired differences. The variation between duplicate filters was ±15% as previously described (Turnbull and Kay, 1976; Turnbull, Evans and Kay, 1977).

### RESULTS

### *Bronchial carcinoma*

The clinical staging and histology of the 31 patients with bronchial carcinoma are shown in Table I. Apart from one individual, all patients were male, and were matched with controls within 10 years of their age. The monocyte chemotactic response of patients with metastatic disease, and their respective controls, are shown in Table I, together with the histology and the treatment being received either at the time of sampling or before the chemotactic assay. There was a significantly greater depression in the monocyte chemotactic response in the metastatic group ($P < 0{\cdot}005$) than in their respective controls. No statistical difference was observed with patients with disease confined to the chest, recurrent cancer or operable disease (Table III, Fig.). It is unlikely that the observed effect with the metastatic group was a result of medication. Three of the 13 were receiving antibiotics and one had treatment with prednisolone, but most of the patients were receiving no treatment at the time of the chemotactic test. The two patients receiving prednisolone in the group with disease confined to the chest had higher chemotactic responses than the control, whereas the one in the metastatic group having corticosteroids had a lower chemotactic response.

TABLE I.—*Clinical Staging and Predominant Histology of the* 31 *Patients Studied with Bronchial Carcinoma*

| | |
|---|---|
| **Clinical stage** | |
| Metastatic | 13 |
| Confined to chest | 12 |
| Recurrent | 2 |
| Operable | 4 |
| **Histology** | |
| Anaplastic | 6 |
| Squamous | 10 |
| Oat cell | 5 |
| Adenocarcinoma | 1 |
| Unknown | 9 |

In the Fig. the results are expressed as the percent migration of each patient's monocytes as compared to their respective matched contol. With the patients as a whole there was a wide scatter; however, the metastatic group responded significantly less in monocyte chemotaxis. The one patient who gave a high response had a pulmonary infection with a white-cell count of 17,000/$\mu$l. There was no significant difference between patients and controls in the other groups, although with operable and recurrent cancer the numbers were very small.

### *Cigarette smokers*

The monocyte chemotactic response of 19 male cigarette smokers, compared with

FIG.—The monocyte chemotactic response of patients with various clinical stages of bronchial carcinoma, and cigarette smokers, expressed as the percent migration of each patient's monocytes compared to its matched control. NS = not significant.

TABLE II.—*The Histology, Treatment and Monocyte Chemotactic Response of Patients with Metastatic Bronchial Carcinoma. Controls were Matched for Age and Sex*

| Pt. No. | Age (Years) Pt. | Age (Years) Control | Histology | Prior treatment (interval between end of treatment and chemotaxis test | Medication at time of test | Monocyte chemotaxis (distance migrated in μm) Pt. | Monocyte chemotaxis (distance migrated in μm) Control |
|---|---|---|---|---|---|---|---|
| 1 | 67 | 69 | Unknown | — | — | 19·7 | 26·8 |
| 2 | 62 | 61 | Anaplastic | — | — | 35·9 | 17·2 |
| 3 | 55 | 59 | Oat cell | PR (22 months) | Prednisolone | 7·6 | 13·3 |
| 4 | 66 | 61 | Unknown | — | Ampicillin | 2·0 | 16·0 |
| 5 | 54 | 60 | Unknown | — | — | 8·0 | 17·0 |
| 6 | 85 | 75 | Unknown | — | — | 2·0 | 18·0 |
| 7 | 73 | 73 | Unknown | — | Ampicillin | 0·5 | 17·0 |
| 8 | 78 | 71 | Unknown | PR (3 years) | — | 8·3 | 23·7 |
| 9 | 56 | 55 | Oat cell | — | — | 26·4 | 33·5 |
| 10 | 61 | 55 | Oat cell | — | — | 13·4 | 33·5 |
| 11 | 53 | 53 | Anaplastic | PR (1 day) | — | 53·6 | 59·0 |
| 12 | 69 | 65 | Squamous | — | Ampicillin | 15·0 | 17·6 |
| 13 | 65 | 65 | Squamous | — | — | 46·4 | 49·7 |

PR = palliative radiotherapy. The chemoattractant was complement-activated serum (2·5%) in Patients 1 to 10 and casein (0·5 mg/ml) in Patients 11 to 13.

non-smoking controls, is shown in Table IV and the Fig. There was no significant difference between the two groups as a whole, nor when the smokers were divided into those who smoked more or less than 20 g per day.

### DISCUSSION

Our results support previous findings on depressed monocyte chemotactic responses in various human cancers (Hausman *et al.*, 1975; Rubin *et al.*, 1976; Boetcher and Leonard, 1974). In the present study on bronchial carcinoma, only those patients with metastatic disease showed a significant depression (Table II, Fig.). Although this may have been a non-specific effect due to general debilitation it was unlikely to be the result of treatment. Many of the patients were receiving no medication at the time of sampling and had not received prior chemotherapy or radiotherapy

TABLE III.—*The Histology, Treatment and Monocyte Chemotactic Response of Patients with Bronchial Carcinoma that was Confined to the Chest, Recurrent or Operable. Controls were Matched for Age and Sex*

| Pt. No. | Age (years) Pt. | Age (years) Control | Histology | Prior treatment (interval since end of treatment until chemotaxis test) | Medication at time of test | Monocyte chemotaxis (distance migrated in µm) Pt. | Monocyte chemotaxis (distance migrated in µm) Control |
|---|---|---|---|---|---|---|---|
| | | | | Confined to chest | | | |
| 1 | 61 | 69 | Anaplastic | PR (20 weeks) | Ampicillin, Prednisolone | 24·0 | 12·7 |
| 2 | 68 | 65 | Squamous | — | Oxytetracycline | 13·9 | 18·4 |
| 3 | 62 | 66 | Anaplastic | — | Ampicillin | 14·0 | 50·2 |
| 4 | 61 | 63 | Squamous | — | — | 16·1 | 17·2 |
| 5 | 60 | 62 | Anaplastic | PR (24 weeks) | — | 17·1 | 48·1 |
| 6 | 64 | 65 | Unknown | — | Prednisolone | 20·7 | 12·9 |
| 7 | 51 | 51 | Oat cell | — | — | 21·1 | 15·2 |
| 8♀ | 54 | 55 | Adenocarcinoma | — | — | 25·9 | 33·5 |
| 9 | 67 | 58 | Oat cell | — | — | 33·0 | 23·6 |
| 10 | 80 | 80 | Squamous | PR (23 days) | — | 37·8 | 59·8 |
| 11 | 68 | 65 | Unknown | Chemotherapy (16 weeks) | — | 36·4 | 46·9 |
| 12 | 57 | 57 | Squamous | Chemotherapy (6 weeks) | — | 58·6 | 50·2 |
| | | | | Recurrent | | | |
| 13 | 82 | 81 | Unknown | — | Oxytetracycline | 42·5 | 30·6 |
| 14 | 53 | 55 | Squamous | — | — | 20·0 | 18·4 |
| | | | | Operable | | | |
| 15 | 65 | 61 | Squamous | — | — | 18·8 | 17·2 |
| 16 | 50 | 48 | Squamous | — | — | 8·0 | 11·0 |
| 17 | 57 | 54 | Anaplastic | Post-surgery (3 weeks) | Ampicillin | 28·0 | 31·0 |
| 18 | 68 | 65 | Squamous | — | Ampicillin | 28·8 | 12·3 |

PR = palliative radiotherapy. The chemoattractant was casein (0·5 mg/ml) in Patients and controls 9 to 12 and 18. In the others it was complement-activated serum (2·5%).

TABLE IV.—*The Monocyte Chemotactic Response of* 19 *Male Cigarette Smokers Compared with Non-smoking Male Controls*

| Age (years) Smoker | Age (years) Control | Tobacco smoked/day (g) | No. of years smoking | Monocyte chemotaxis (mean cell count) Smoker | Monocyte chemotaxis (mean cell count) Control |
|---|---|---|---|---|---|
| 56 | 63 | 60 | 41 | 49 | 76 |
| 62 | 51 | 60 | 45 | 45 | 53 |
| 49 | 42 | 53 | 31 | 65 | 61 |
| 42 | 33 | 40 | 17 | 76 | 53 |
| 40 | 55 | 36 | 19 | 66 | 74 |
| 52 | 64 | 30 | 38 | 116 | 90 |
| 49 | 31 | 30 | 35 | 17 | 38 |
| 58 | 44 | 26 | 40 | *46 | 31 |
| 64 | 60 | 23 | 47 | 57 | 48 |
| 27 | 22 | 21 | 8 | 82 | 81 |
| 35 | 34 | 20 | 16 | 43 | 70 |
| 48 | 54 | 20 | 35 | 76 | 95 |
| 32 | 28 | 20 | 14 | 37 | 28 |
| 25 | 27 | 20 | 8 | 84 | 76 |
| 47 | 34 | 20 | 23 | 84 | 66 |
| 28 | 23 | 16 | 10 | 134 | 72 |
| 54 | 55 | 15 | 36 | 65 | 100 |
| 29 | 29 | 29 | 5 | 75 | 91 |
| 53 | 49 | 14 | 28 | 50 | 59 |

The chemoattractant was 2·5% CVF-activated serum, with the exception of the pair indicated (*) in which the concentration was 1·25%.

(Tables II and III). In a similar study on malignant melanoma (Rubin *et al.*, 1976), only patients with advanced disease had a monocyte defect.

The inhibitor of macrophage chemotaxis produced by various transplanted neoplasms in the peritoneal cavity of mice was partially identified as a protein of mol. wt. 6000–10,000 (Snyderman and Pike, 1976). A similar inhibitor is possibly elaborated from human neoplasms and if it is related to tumour mass this may account for the effect observed in the metastatic group in the present study.

A recent leading article in the *Lancet* (1976), discussing the possible role of macrophages in tumour surveillance, emphasized the present difficulties in relating *in vitro* data from man and experimental animals to the clinical situation. Nevertheless, if tumour-derived material with inhibitory effects on monocyte function can be demonstrated, this may provide some additional evidence to support the concept that tumour products overcome possible tumoricidal effects of mononuclear phagocytes. Experiments currently in progress suggest that extracts of human tumours may inhibit the chemotactic response of normal blood monocytes (Abell, C. and Kay, A. B., unpublished).

Cigarette smokers showed no difference from controls in their monocyte migratory capacity, indicating that monocyte chemotaxis will not be useful in detecting individuals at risk for developing bronchial carcinoma (Table IV). Studies with human alveolar macrophages obtained from smoking and non-smoking volunteers demonstrated both an increase in the number of cells recovered by bronchial lavage and of the chemotactic response of these cells from smokers when compared to controls (Warr and Martin, 1974). This suggests that cigarette smoke probably has an initial non-specific "macrophage-activating effect" analogous to the influx of macrophages into tissues treated with various irritants such as mineral oil and glycogen.

The chemotactic activity of human serum activated with cobra venom factor is due almost entirely to the fragment cleaved from the 5th component of complement (C5a) liberated as a result of activation of the alternate pathway. When CVF-activated serum is placed on either side of the micropore chamber, migration is either minimal or absent, suggesting that this agent evokes chemotaxis, (i.e. directional migration) rather than random migration (Kay, unpublished).

There are difficulties in employing the chemotactic assay for clinical studies. The reasons include possible deterioration of the chemoattractant during storage, variations in an individual's cell response with time, and failure to reproduce this biological assay exactly on each occasion. In the present study these difficulties were largely overcome by matching each patient or smoker with a control individual for age and sex, withdrawing blood from each pair at the same time and performing the test under identical conditions. Comparison of these matched pairs by the Wilcoxon test of paired differences allowed statistical analysis.

Thus the present study suggests that defects in monocyte chemotaxis are only apparent at advanced stages of bronchial carcinoma, and not in those with relatively confined disease or in those individuals who are at risk for developing bronchial neoplasms.

We wish to acknowledge the excellent technical assistance of Mrs Elaine Soutar. This work was supported by the Cancer Research Campaign. We are grateful to the following clinicians who allowed us to obtain blood samples from patients under their charge: Professor J. W. Crofton, Dr A. C. Douglas, Mr R. J. M. McCormack, Dr G. J. R. McHardy, Dr B. H. R. Stack and Mr P. R. Walbaum.

### REFERENCES

Alexander, P. (1976) Surveillance against Anaplastic Cells. Is it mediated by Macrophages? *Br. J. Cancer*, **33**, 344.

BALLOW, M. & COCHRANE, C. G. (1969) Two Anticomplementary Factors in Cobra Venom: Hemolysis of Guinea Pig Erythrocytes by One of Them. *J. Immunol.*, **103**, 944.

BOETCHER, D. A. & LEONARD, E. J. (1974) Abnormal Monocyte Chemotaxis Response in Cancer Patients. *J. natn. Cancer Inst.*, **52**, 1091.

BÖYUM, A. (1968) Isolation of Leucocytes from Human Blood. Further Observations. Methylcellulose, Dextran, and Ficoll as Erythrocyte aggregating Agents. *Scand. J. clin. Lab. Invest.*, **21** (Suppl. 97), 31.

HAUSMAN, M. S., BROSMAN, S., SNYDERMAN, R., MICKEY, M. R. & FAHEY, J. (1975) Defective Monocyte Function in Patients with Genitourinary Carcinoma. *J. natn. Cancer Inst.*, **55**, 1047.

HIBBS, J. B., JR., LAMBERT, L. H., JR. & REMINGTON J. S. (1972) Control of Carcinogenesis: A Possible Role for the Activated Macrophage. *Science, N.Y.*, **177**, 998.

*Lancet* (1976) Macrophages v. Cancer. **ii**, 27.

RUBIN, R. H., COSIMI, A. B. & GOETZL, E. J. (1976) Defective Human Mononuclear Leukocyte Chemotaxis as an Index of Host Resistance to Malignant Melanoma. *Clin. Immunol. Immunopathol.*, **6**, 376.

SNYDERMAN, R., ALTMAN, L. C., HAUSMAN, M. S. & MERGENHAGEN, S. E. (1972) Human Mononuclear Leukocyte Chemotaxis: A Quantitative Assay for Humoral and Cellular Chemotactic Factors. *J. Immunol.*, **108**, 857.

SNYDERMAN, R. & PIKE, M. C. (1976) An Inhibitor of Macrophage Chemotaxis produced by Neoplasms. *Science, N.Y.*, **192**, 370.

TURNBULL, L. W. & KAY, A. B. (1976) Eosinophils and Mediators of Anaphylaxis. Histamine and Imidazole Acetic Acid as Chemotactic Agents for Human Eosinophil Leucocytes. *Immunology*, **31**, 797.

TURNBULL, L. W., EVANS, D. P. & KAY, A. B. (1977) Human Eosinophils, Acidic Tetrapeptides (ECF-A) and Histamine. Interactions *in vitro* and *in vivo*. *Immunology*. **32**, 57.

WARR, G. A. & MARTIN, R. R. (1974) Chemotactic Responsiveness of Human Alveolar Macrophages: Effects of Cigarette Smoking. *Infect. Immunity*, **9**, 769.

ZIGMOND, S. H. & HIRSCH, J. G. (1973) Leukocyte Locomotion and Chemotaxis. New Methods for Evaluation and Demonstration of Cell-derived Chemotactic Factor. *J. exp. Med.*, **137**, 387.


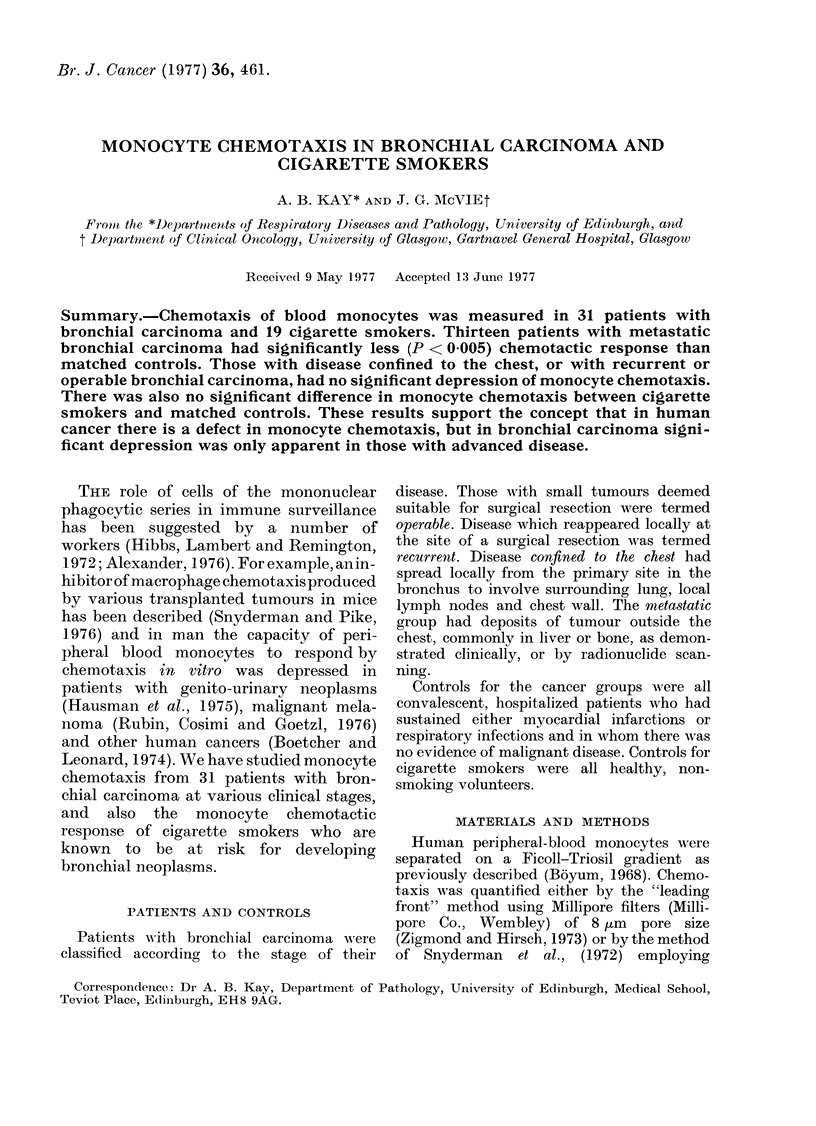

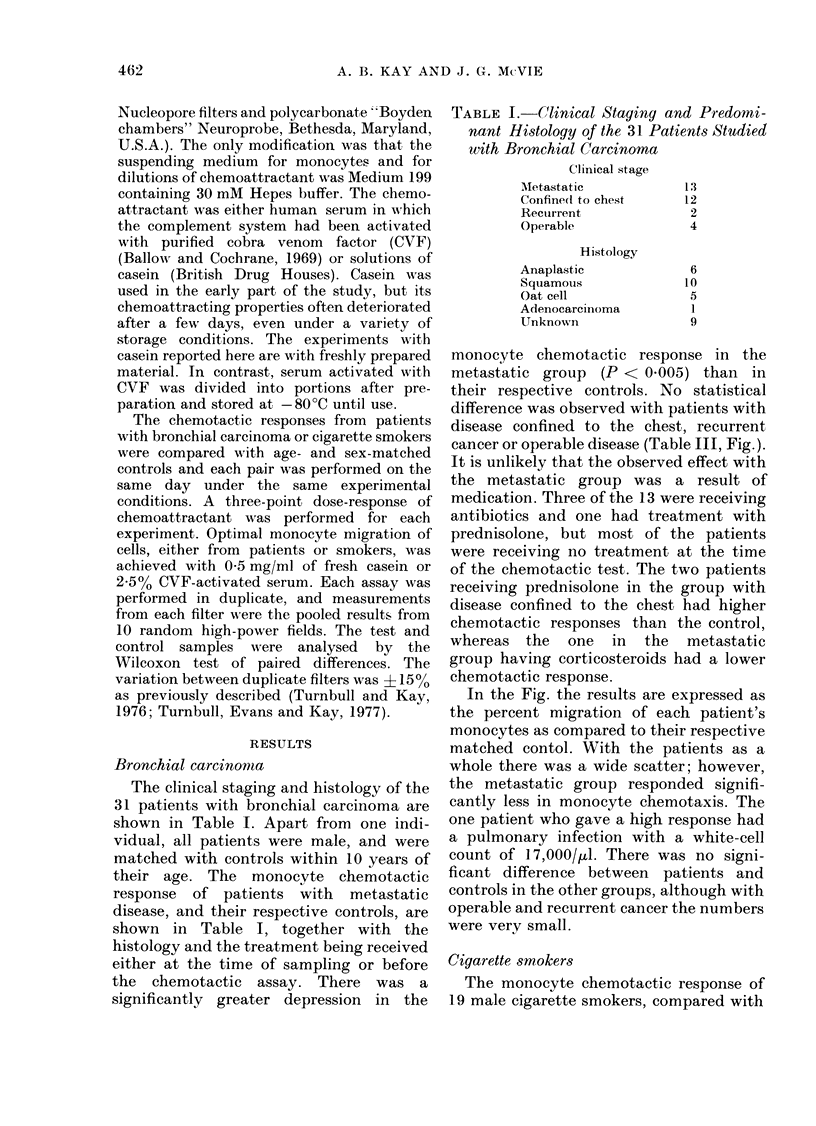

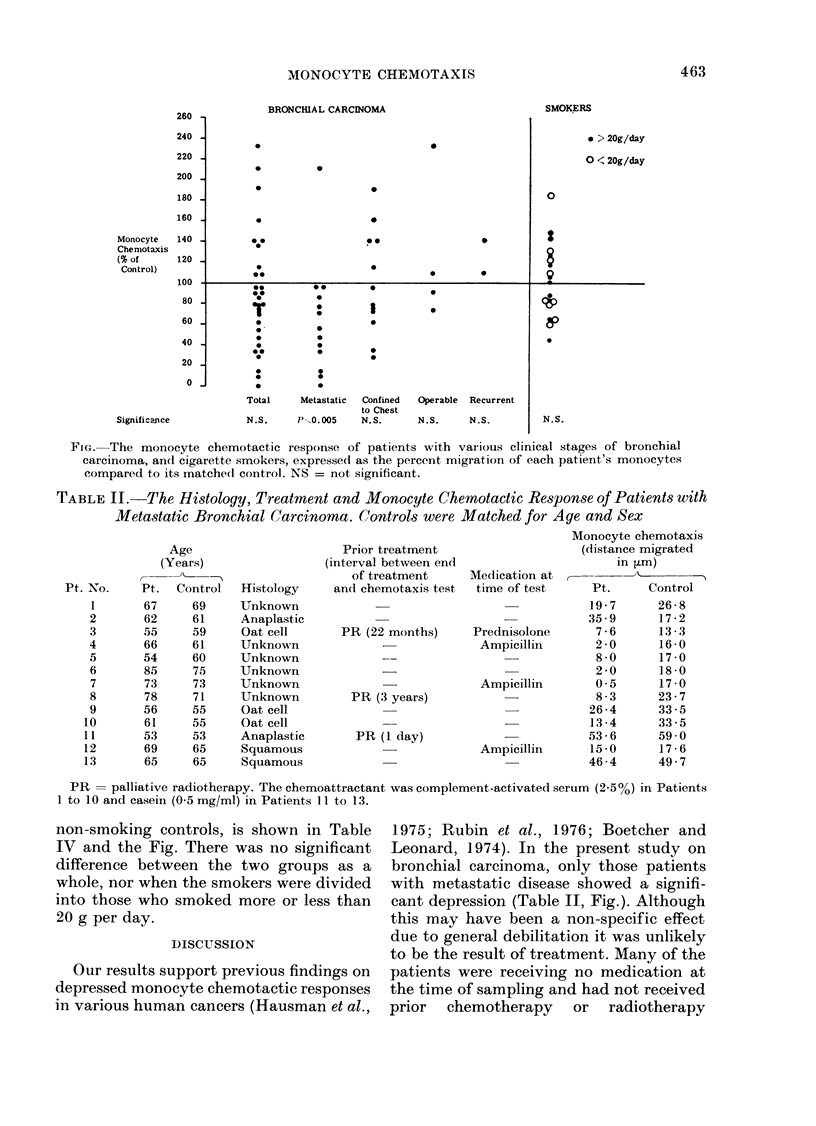

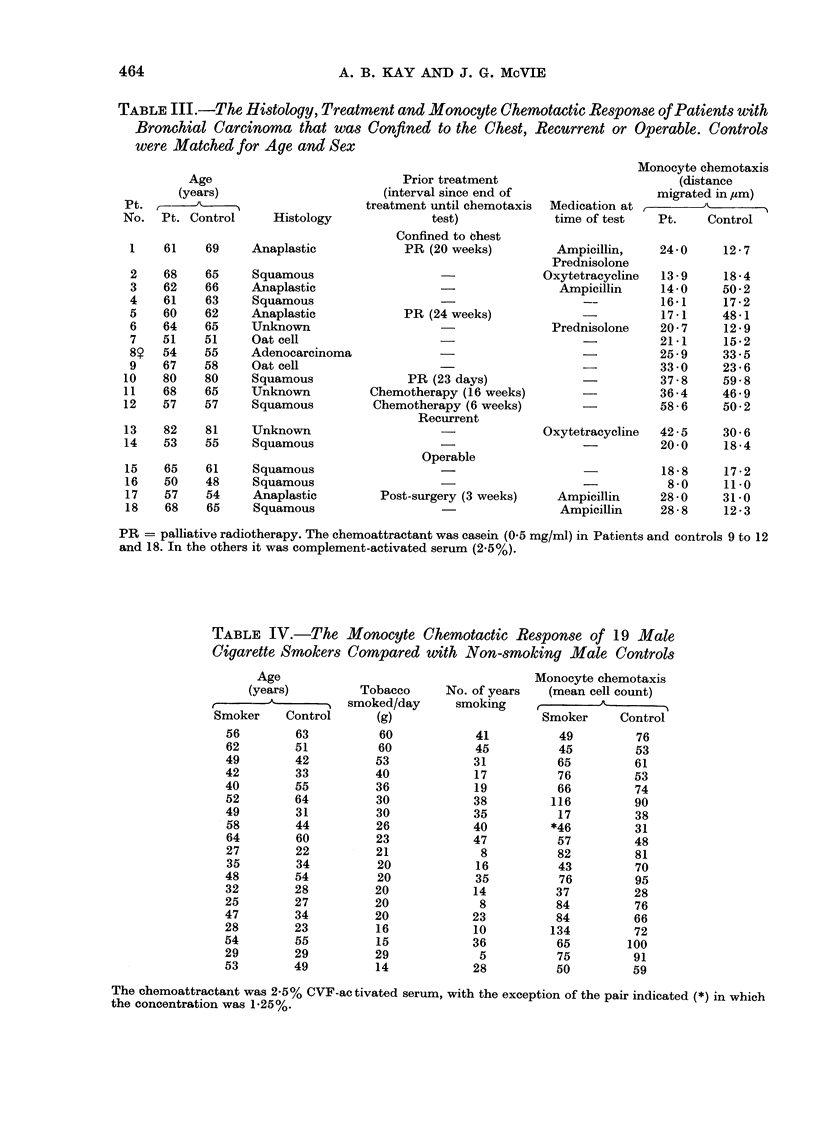

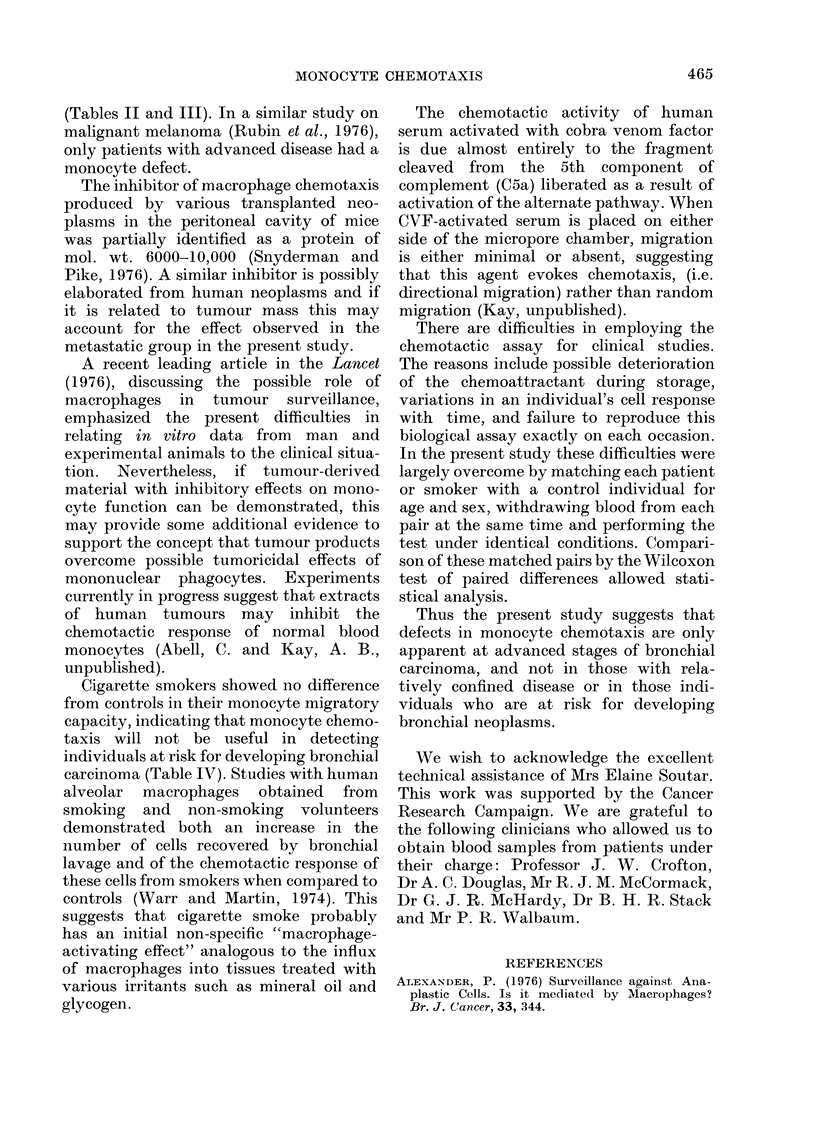

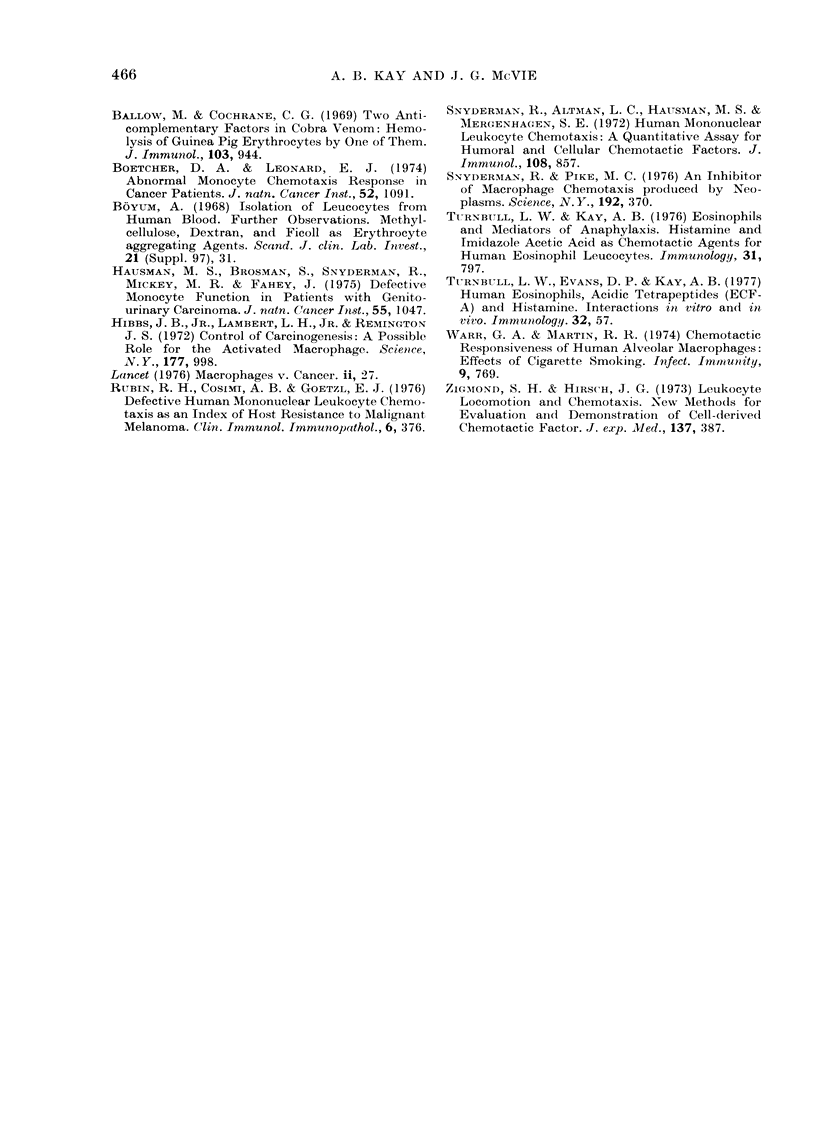


## References

[OCR_00818] Alexander P. (1976). Letter: Surveillance against neoplastic cells-is it mediated by macrophages?. Br J Cancer.

[OCR_00825] Ballow M., Cochrane C. G. (1969). Two anticomplementary factors in cobra venom: hemolysis of guinea pig erythrocytes by one of them.. J Immunol.

[OCR_00831] Boetcher D. A., Leonard E. J. (1974). Abnormal monocyte chemotactic response in cancer patients.. J Natl Cancer Inst.

[OCR_00874] (1976). Eosinophils and mediators of anaphylaxis. Histamine and imidazole acetic acid as chemotactic agents for human eosinophil leucocytes.. Immunology.

[OCR_00843] Hausman M. S., Brosman S., Snyderman R., Mickey M. R., Fahey J. (1975). Defective monocyte function in patients with genitourinary carcinoma.. J Natl Cancer Inst.

[OCR_00848] Hibbs J. B., Lambert L. H., Remington J. S. (1972). Control of carcinogenesis: a possible role for the activated macrophage.. Science.

[OCR_00856] Rubin R. H., Cosimi A. B., Goetzl E. J. (1976). Defective human mononuclear leukocyte chemotaxis as an index of host resistance to malignant melanoma.. Clin Immunol Immunopathol.

[OCR_00862] Snyderman R., Altman L. C., Hausman M. S., Mergenhagen S. E. (1972). Human mononuclear leukocyte chemotaxis: a quantitative assay for humoral and cellular chemotactic factors.. J Immunol.

[OCR_00869] Snyderman R., Pike M. C. (1976). An inhibitor of macrophage chemotaxis produced by neoplasms.. Science.

[OCR_00881] Turnbull L. W., Evans D. P., Kay A. B. (1977). Human eosinophils, acidic tetrapeptides (ECF-A) and histamine. Interactions in vitro and in vivo.. Immunology.

